# The Role of Perineuronal Nets in the Contralateral Hemisphere in the Electroacupuncture-Mediated Rehabilitation of Poststroke Dysphagia Mice

**DOI:** 10.1523/ENEURO.0234-23.2023

**Published:** 2023-12-01

**Authors:** Si Yuan, Jiahui Shi, Xiaorong Tang, Bing Deng, Zhennan Wu, Bo Qiu, Shumin Lin, Chang Ji, Lin Wang, Shuai Cui, Nenggui Xu, Lulu Yao

**Affiliations:** 1 South China Research Center for Acupuncture and Moxibustion, Medical College of Acu-Moxi and Rehabilitation, Guangzhou University of Chinese Medicine, Guangzhou, Guangdong Province 510006, China; 2 Department of Rehabilitation of Traditional Chinese Medicine, Hunan University of Chinese Medicine, 410208, Changsha, Hunan Province, China; 3 The First Affiliated Hospital, Jinan University, Guangzhou, Guangdong Province 510630, China; 4 Research Institute of Acupuncture and Meridian, College of Acupuncture and Moxibustion, Anhui University of Chinese Medicine, Hefei, Anhui Province 230012, China

**Keywords:** PNNs, post-stroke dysphagia, contralateral primary motor cortex, electroacupuncture, swallowing function

## Abstract

Acupuncture at Lianquan (CV23) acupoint has been shown to improve swallowing function in poststroke dysphagia (PSD). This improvement is supposed to be associated with the regulation of neuronal activity in the contralateral primary motor cortex (M1), while the underlying mechanism still needs to be elucidated. Perineuronal nets (PNNs) are well-known to be involved in the regulation of neuronal activity. Thus, we here aimed to detect the role of PNNs in the contralateral M1 hemisphere in the electroacupuncture (EA)-mediated effect in male mice. The results were obtained from a combination of methods, including *in vitro* slice electrophysiological recording, *in vivo* electrophysiological recording, and immunofluorescent staining in male mice. These results showed a decrease of the excitatory postsynaptic currents (sEPSCs) and no alteration of the inhibitory postsynaptic currents (sIPSCs) in the GABAergic neurons and the tonic inhibition in the excitatory neurons in the contralateral M1 after stroke induction, and EA recovered the impaired sEPSCs in the GABAergic neurons. We further found that the effect of EA-induced increase of c-Fos expression, enhancement of spike firing, potentiation of sEPSCs in the excitatory neurons, and improvement of swallowing function were all blocked by the removal of PNNs in the contralateral M1. In conclusion, the PNNs in the contralateral M1 was suggested to be participated in stroke pathogenesis and might be associated with the EA-mediated swallowing function rehabilitation of PSD in male mice. Our study provides insight into how PNNs might be involved in the mechanism of EA treatment for stroke rehabilitation.

## Significance Statement

In the present study, we concluded that the effect of electroacupuncture (EA)-mediated the regulation of neuronal activity and swallowing function was absent after perineuronal nets (PNNs) degradation in the contralateral primary motor cortex (M1). The PNNs in the contralateral M1 was associated with the stroke pathogenesis and might be participated in the EA-mediated rehabilitation of poststroke dysphagia (PSD) mice, and whether PNNs involved in the regulation of EA-mediated swallowing function in PSD mice still needs to be explored. Our study provides insight into how the PNNs might be involved in the mechanism of EA treatment for stroke rehabilitation.

## Introduction

Stroke is one of the leading causes of disability and death worldwide (Katan and Luft, 2018), and the prevalence of dysphagia is up to 80% (González-Fernández et al., 2013; Sugiyama et al., 2014; Rofes et al., 2018). Traditional therapies of dysphagia following stroke focus on compensations and behavioral rehabilitation strategies (Jones et al., 2020). Electroacupuncture (EA), as an alternative and supplementary therapy for stroke recovery, has been more and more widely practiced (Han et al., 2020). In clinical practice, EA at Lianquan (CV23), located at the upper margin of the midline of the mandible (Yuan et al., 2022; L. Yao et al., 2023), was demonstrated to promote swallowing function, improve the quality of life, and reduce the incidence of dysphagia related complications, including pneumonia, undernutrition, and psychological and social interaction disorders (Li et al., 2021). In animals, EA has been shown to activate the swallowing-related neurons in the contralateral primary motor cortex (M1; Cui et al., 2020; S. Yao et al., 2020; L. Yao et al., 2023). However, how the neuronal activity in the contralateral M1 is regulated by EA still needs to be elucidated.

Perineuronal nets (PNNs) are a highly condensed form of the extracellular matrix (ECM) in the CNS (Fawcett et al., 2019), which surrounds the soma, dendrites, and proximal axons of neurons (Karetko-Sysa et al., 2011). A distinctive feature of the PNNs is a reticular structure resulting from the aggregation of ECM molecules rich in chondroitin sulfate proteoglycans (CSPGs; Galtrey and Fawcett, 2007; Miao et al., 2014; Quattromani et al., 2018a). PNNs is an important regulator of excitability and synaptic plasticity (Sitaš et al., 2022). Understanding the functions of PNNs may be essential for characterizing the mechanisms of altered cortical excitability observed in neurodegenerative and neurodevelopmental disorders, including epilepsy, stroke, schizophrenia, and Alzheimer’s disease (Galtrey and Fawcett, 2007; Enwright et al., 2016; Quattromani et al., 2018a; Wen et al., 2018). Previous study suggested that degradation of PNNs could enhance neuronal activity and plasticity after CNS injury (Soleman et al., 2013). In the stroke mouse model, downregulating PNNs could reduce the neurite outgrowth in an inhibitory environment, reactivate plasticity, and thus promote functional recovery (Dzyubenko et al., 2018; Quattromani et al., 2018b). Regulation of contralateral M1 excitability is helpful for stroke recovery (Tang et al., 2022). Our previous study showed that EA at CV23 increased neuronal activity in the contralateral M1 (S. Yao et al., 2020; L. Yao et al., 2023), and EA may affect the PNNs and promote the recovery of the motor function (Hu et al., 2020). Therefore, we hypothesized that the PNNs in the contralateral M1 might be involved in regulating neuronal activity during the EA treatment for stroke recovery.

In this study, we aimed to examine the role of PNNs in the contralateral M1 in the EA-mediated improvement of swallowing function in the poststroke dysphagia (PSD). The results demonstrated that the mice with PNNs degradation in the contralateral hemisphere showed no impaired neuronal activity represented by c-Fos expression, spike firing *in vivo*, and synaptic transmission *in vitro*, and no dysfunctional swallowing process after stroke, and EA-mediated effect also disappeared.

## Materials and Methods

### Animals

All experiments were performed using two- to three-month-old male C57BL/6J mice obtained from the Animal Laboratory Center of Guangzhou University of Chinese Medicine [license No. SCXK (Yue) 2018-0034]. GAD67-GFP knock-in mice were from Professor Yongjun Chen (Ting et al., 2011). A total number of 143 C57BL/6J mice and a total number of 17 GAD67- GFP mice were used in this study as follow, and see the table below for details in the experimental list (Table 1). Abbreviations were found in Table 2. Animals were kept in the room with a regular inverse 12/12 h light/dark cycle in groups of no more than five animals per cage. Experimental procedures were conducted by the National Institutes of Health *Guide for the Care and Use of Laboratory Animals* and approved by the Committee for Care and Use of Research Animals of Guangzhou University of Chinese Medicine.

**Table 1:** Experimental list

Figures	Purpose	Experiments	Number of mice
Figure 1*A–F*	To assess the effect of stroke and EA treatment on the synaptic activity	Electrophysiological recording in slices	GAD67-GFP mice: Sham group (*N* = 7), Stroke group (*N* = 5), and Stroke + EA group (*N* = 5)
Figure 1*G,H*	To validated whether the tonic inhibition was changed	Electrophysiological recording in slices	9/3*
Figure 2*A–G*	To explore the expressions of PNNs in different conditions and show the injection sites of PNNs degradation and test the efficacy of injection of P-nase or ChABC	(1) Injection of P-nase or ChABC (2) Immunofluorescence	P-nase-injected group (*N* = 2) ChABC-injected group (*N* = 2) 27/3*
Figure 3*A–C*	To explore the role of PNNs in the regulation of neuronal activity in the stroke or EA treatment	(1) Injection of P-nase or ChABC (2) Immunofluorescence	18/3*
Figure 4*A–E*	To explore the role of PNNs in the regulation of neuronal activity in the stroke or EA treatment	(1) Injection of P-nase or ChABC (2) *In vivo* electrophysiological recording	24/4*
Figure 5*A–F*	To record the sEPSCs and sIPSCs	(1) Injection of P-nase (2) Electrophysiological recording in slices	Sham group (*N* = 6), Stroke group (*N* = 4), and Stroke + EA group (*N* = 3)
Figure 5*A–F*	To record the sEPSCs and sIPSCs	(1) Injection of ChABC (2) Electrophysiological recording in slices	Sham group (*N* = 6), Stroke group (*N* = 3) and Stroke + EA group (*N* = 3)
Figure 6*A,B*	To validate the function of PNNs in the EA-mediated improvement of swallowing function	(1) Injection of P-nase or ChABC (2) Electromyography recording in the mylohyoid *in vivo*	36/6*
Total = 160 mice, GAD67-GFP = 17 mice, C57 = 143 mice			

**Table 2:** Abbreviations

Full name	Abbreviation
Analysis of variance	ANOVA
ChondroitinaseABC	ChABC
Central nervous system	CNS
Chondroitin sulfate proteoglycans	CSPGs
Lianquan	CV23
Electroacupuncture	EA
Extracellular matrix	ECM
Electromyography	EMG
Interspike interval	ISI
Primary motor cortex	M1
Neuronal activity-regulated pentraxin	Narp
Nogo receptor	NgR
Optimal cutting temperature	OCT
Orthodenticle homeobox protein 2	OTX2
Phosphate buffer	PB
Phosphate buffered saline	PBS
Paraformaldehyde	PFA
Penicillinase	P-nase
Perineuronal nets	PNNs
Poststroke dysphagia	PSD
Parvalbumin neurons	PV^+^
Semaphorin3A	Sema3A
Excitatory postsynaptic currents	sEPSCs
Inhibitory postsynaptic currents	sIPSCs
2,3,5-Triphenyltetrazolium chloride	TTC
*Wisteria floribunda* agglutinin	WFA

### Establishing a poststroke dysphagia mouse model

The methods have been previously described (Cui et al., 2020; S. Yao et al., 2020; Yuan et al., 2022; L. Yao et al., 2023). Briefly, animals were anesthetized by intraperitoneal injection of avertin solution (1.25%, Sigma-Aldrich) with an injection dose of 20 ml/kg. To induce photochemical stroke, rose bengal (1.5%, Sigma-Aldrich) of 10 ml/kg was injected intraperitoneally ∼10 min before exposing the skull. To irradiate the target site, the selective area was determined by using bregma and lambda as a point of reference in the Paxinos and Franklin’s *Mouse Brain Atlas* (fifth edition) (Reinitz et al., 2016). After about 10 min, photoactivation was initiated using a laser focused on the brain region that is located on the right M1 coordinates (AP: −0.12 mm, M/L: +1.03 mm). The parameter of irradiation was set as the wavelength at 532 nm and power at 15 mW as described before. The Stroke group were exposed to laser irradiation 7 min after rose bengal administration, but the Sham group injected with rose bengal, and the skin was cut, following the skull was exposed without laser irradiation.

### Electroacupuncture treatment

EA treatment was performed as described previously (Yuan et al., 2022; L. Yao et al., 2023). After routinely sterilizing the neck skin, EA was applied at “Lianquan (CV23)” for 15 min. The acupoint of CV23 located on the upper margin of the midline of the mandible (L. Yao et al., 2023). The needles were inserted into the acupoint at an angle of 15∼45° with an ∼5-mm depth at CV23. One electrode in one pair of EA instruments (HANS-200A/100B; HANS) was connected to the EA needle on the CV23 acupoint, and the other electrode was inserted 2 mm adjacent to CV23 acupoint. The parameters of EA treatment were set as an intensity of 1 mA and a frequency of 2 Hz for 15 min, and this stimulation could induce visible muscle contractions. EA stimulation was treated only once.

### Stereotactic injection of ChABC or P-nase

On the day of injecting chondroitinaseABC (ChABC) or penicillinase (P-nase), animals were anesthetized with an intraperitoneal injection of avertin solution (1.25%, Sigma-Aldrich) with an injection dose of 20 ml/kg. To disrupt PNNs locally in the M1, adult mice underwent a stereotaxic injection of the bacterial enzyme chondroitinaseABC (ChABC). ChABC (40 U/ml, Sigma-Aldrich) was diluted in saline. A volume of 300 nl of solution at 30 nl/min was injected into the M1 contralateral to the ischemic regions (A/P: −0.12 mm, M/L: −1.03 mm, D/V: −1.1 mm). In the penicillinase (P-nase) group, P-nase (40 U/ml, Sigma-Aldrich), a bacterial enzyme lacking endogenous substrate, as a widely used control compound for ChABC (Gherardini et al., 2015), was administered as the same procedure. The skin was sutured and the mouse was gently removed from the frame and kept at 37°C in a heated chamber until full recovery.

### *In vitro* slice electrophysiological recording

**Figure 1.**
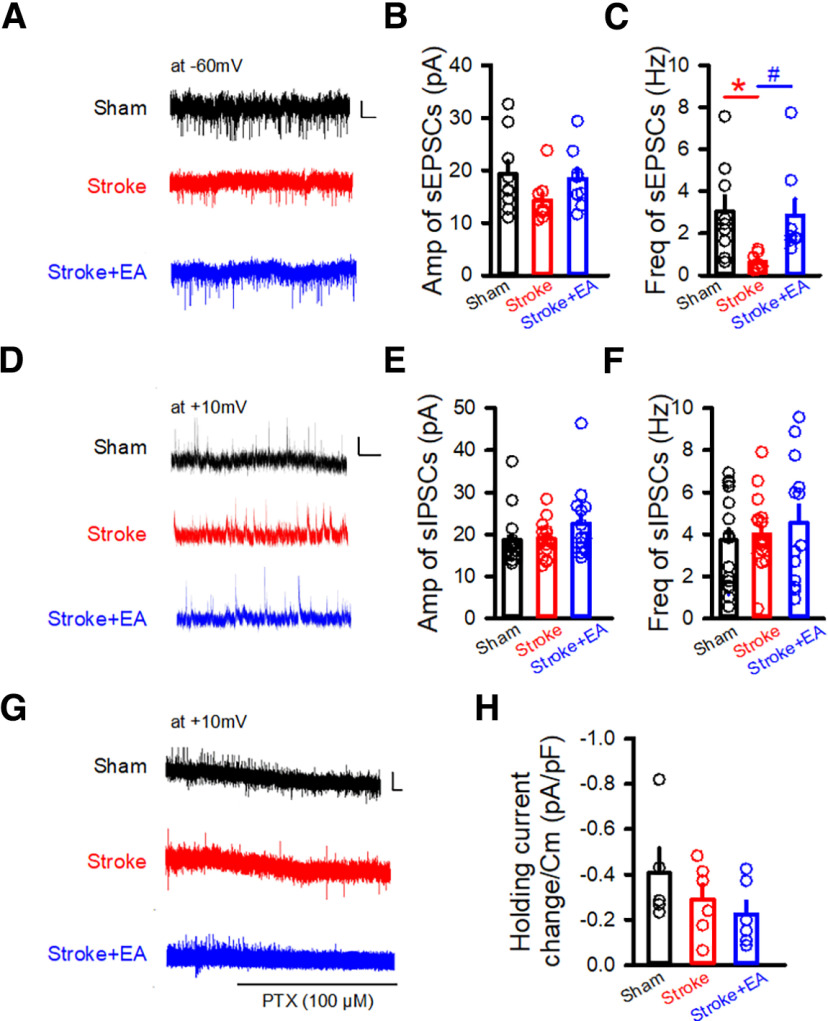
sEPSCs in the GABAergic neurons and tonic inhibition in the excitatory neurons in the contralateral M1 were recorded by *in vitro* slice electrophysiological recording. ***A***, Sample traces showing sEPSCs (clamped at −60 mV) recorded in the GABAergic neurons fromGAD67-GFP transgenic mice in the Sham, Stroke, and Stroke + EA groups. Scale bars: 2 s, 5 pA. ***B***, ***C***, Quantifications of amplitude (Amp) and frequency (Freq) in the contralateral M1 from the Sham, Stroke, and Stroke + EA groups. The amplitude of sEPSCs was not affected by stroke or EA treatment (***B***). While there is a significant decrease in the frequency of sEPSCs in the Stroke group compared with that in the Sham group, and this impairment could be improved by EA treatment (***C***). *N* (cells) = 8–9 per group. *N* (mice) = 7, 5, and 5 for the Sham, Stroke, and Stroke + EA groups, respectively. Compared with Sham group, *p <* *0.05; compared with Stroke group, #*p* <0.05. ***D***, Sample traces showing sIPSCs (clamped at +10 mV) recorded in the GABAergic neurons from GAD67-GFP transgenic mice in the Sham, Stroke, and Stroke + EA groups. Scale bars: 1 s, 20 pA. ***E***, ***F***, Quantifications of amplitude (Amp) and frequency (Freq) in the contralateral M1 from the Sham, Stroke, and Stroke + EA groups. The amplitude and frequency of sIPSCs was not affected by stroke or EA treatment. *N* (cells) = 13–17 per group. *N* (mice) = 7, 5, and 5 for the Sham, Stroke, and Stroke + EA groups, respectively. ***G***, Sample traces showing the recorded tonic inhibition by changes of holding current induced by picrotoxin (PTX; 100 μm) in the excitatory neurons clamped at +10 mV. Scale bars: 4 s,10 pA. ***H***, The tonic inhibition, calculated as the change of holding current after PTX application divided by the capacitance, showed no significant difference among three groups. *N* (cells) = 6 per group. *N* (mice) = 3 per group. Data are shown as mean ± SEM. One-way ANOVA.

To target the GABAergic neurons, the GAD67-GFP transgenic mice were used (Fig. 1*A–F*). The ChABC or P-nase was injected in the M1 1 d before the stroke. About 24 h after modeling, mice were anesthetized using avertin solution and decapitated. The brain was quickly decapitated and placed in the ice-cold cutting solution [artificial CSF (aCSF)], including (in mm): 110 choline chloride, 7 MgSO_4_, 2.5 KCl, 1.25 NaH_2_PO_4_, 25 NaHCO_3_, 25 D-glucose, 11.6 sodium ascorbate, 3.1 sodium pyruvate, and 0.5 CaCl_2_ and gassed with 95% O_2_ and 5% CO_2_. Only the left hemisphere of the brain was kept for cutting and further recording, and the right hemisphere was cut off and discarded. Coronal frontal sections (400 μm) were cut on a VT1200S vibratome (Leica). Brain slices were allowed to recover for ∼30 min at ∼32°C, and then transferred to a holding chamber for incubation at room temperature (23–26°C) containing (in mm): 127 NaCl, 2.5 KCl, 1.25 NaH_2_PO_4_, 25 NaHCO_3_, 25 D-glucose, 2 CaCl_2_, and 1 MgSO_4_.

Individual slice was transferred to the recording chamber on an upright microscope (Eclipse FN1, Nikon) with a ×40 water-immersion differential interference contrast objective, and screened on Digital CMOS camera (C11440-42U, Hamamatsu). Slices were constantly perfused at room temperature with oxygenated aCSF (4–5 ml/min). Recordings were made from M1 neurons located in layers 5/6. Neurons were clamped at −60 mV to record the sEPSCs and at +10 mV to record the sIPSCs. To record the tonic inhibition, the neurons were clamped at +10 mV, the stable baseline was obtained at least 5 min and then the picrotoxin (100 μm) was applied (Wlodarczyk et al., 2013). The internal solution was containing as follows (in mm): 125 CsMeSO_4_, 5 NaCl, 1.1 EGTA, 10 HEPES, 0.3 Na_2_GTP, 4 Mg-ATP, and 5 QX-314. Pyramidal neurons characterized with apical dendrite and triangular somata were selected for recording. Data were acquired using a Multiclamp 700B amplifier and a Digidata 1550B (Molecular Devices). The sampling rate was set as 10 kHz and signals filtered at 2 kHz. If series resistance was over 30 MΩ, the data of the recorded neurons were excluded.

### *In vivo* electrophysiological recording

All mice were anesthetized with 1−2% isoflurane with an oxygen/air mixture and then fixed on the brain stereotactic apparatus (RWD). A gap was made in the middle of the scalp and the skull was exposed. The target area was located, and the holes were drilled by the skull drill. Electrodes were then inserted into M1 (in the left hemisphere; A/P: −0.12 mm, M/L: −1.03 mm, D/V: 0.8–1.10 mm). At the same time, an annular tube was fixed with dental cement in the right M1 for modeling. Eight matrix electrodes were used to record spontaneous discharges of M1 L5/6 using a multichannel recording system (Plexon Inc.). Spikes were recorded for 5 min in each group. An offline sorter (Plexon Inc.) was used to filter the signal and the processed signals were analyzed using NeuroExplorer (Nex Technologies). Units were categorized as a neuronal cluster with similar firing properties. Neurons were identified by autocorrelation analysis. The characteristics of putative excitatory neurons was low mean discharge frequency and irregular discharge pattern, the short interspike interval (ISI) was dominant, and exponential attenuation was present after 3- to 5-ms ISI, wide waveform. The characteristics of putative inhibitory neurons was high mean discharge frequency, delayed spikes, and slower attenuation and narrow waveform.

### Immunofluorescence

Animals were perfused transcardially, with 0.9% saline and then with paraformaldehyde (PFA) 4% in 0.1 m phosphate buffer (PB). The brains were extracted, stored in 4% PFA at 4°C overnight, and cryopreserved in 15% and 30% sucrose in PB. Brains were then embedded in the Tissue-Tek optimal cutting temperature (OCT) compound (Sakura Finetek). Then brains were cut into 40-μm sections using a sliding freezing microtome (Leica CM1950). Free-floating slices were then transferred to 0.1 m PBS wells at 4°C. Slices were washed 3 × 10 min in 0.1 m PBS, followed by incubated for 1 h in blocking solution (1% BSA and 0.3% Triton X-100 in PBS) and incubated at 4°C with primary antibodies (see below for a full list of antibodies) in blocking solution.

For the PNNs staining, slices were incubated with the primary antibody for 12 h at 4°C. For the c-Fos staining, slices were incubated with the primary antibody for 24 h at 4°C. The Sections were washed three times in 1× PBS for 5 min and incubated for 2 h at room temperature with secondary antibodies (see below for a full list of antibodies) in 0.3% Triton X-100 in PBS. Sections were then washed three times in 1× PBS for 5 min. Finally, the stained tissue was mounted on glass slides with Fluoroshield with 4,6-diamidino-2-phenylindole (DAPI; 1 μg/5 ml, Sigma) and mounted on slides with a mounting medium (50% Glycerol anhydrous in PBS, Biofroxx). The sections were imaged with a confocal microscope (Nikon). Confocal fluorescence images were acquired on a Nikon scanning laser microscope using 20× or 40× air objectives.

### Antibodies

The following primary antibodies were used: lectin from *Wisteria floribunda* (Sigma-Aldrich, L8258; diluted 1:500), Rabbit anti-c-Fos (Cell Signaling Technology, catalog #2250; diluted 1:500). The following secondary antibodies were used, all from streptavidin (conjugated to Alexa Fluor 488, S11223 diluted 1:500), donkey anti-rabbit (conjugated to Alexa Fluor 488, catalog #711-545-152; diluted 1:500).

### Image analysis

ImageJ was used to count the cells expressing c-Fos and PNNs in brain sections. The investigator was blinded to groups for c-Fos and PNNs identification and counting by using a brain atlas for guidance. For the density of c-Fos and PNNs, images were acquired using a 20× or 40× objective, and the area was processed using ImageJ software (1.52a; National Institutes of Health). The density of c-Fos-positive cell was calculated for each slice by dividing the total sampled cell numbers by the total area of the region.

### Electromyography recording in the mylohyoid *in vivo*

In the previous studies, the PSD mice were determined by the results obtained from electromyography (EMG) recording and water consumption test simultaneously (Cui et al., 2020; Yuan et al., 2022; L. Yao et al., 2023). The results showed that the EMG responses was impaired along with decreased water consumption in the stroke model group (Cui et al., 2020). In the study, we detected the swallowing function by EMG recording. The mice were anesthetized with 4% isoflurane (Sigma-Aldrich). The mice were supine and fixed on the stereotaxator. A syringe with 10 ml of pure water was placed in a microinjection pump (Stoelting), and an aqueduct was placed under the tongue of the mouse. Recording electrodes were inserted into the mylohyoid muscle. After anesthesia, water stimulated the myoelectric activity of hyoid muscle (2 μl/s, 10 s), which was recorded by Spike2 software (CED). The data were digitized at 5 kHz using a Power 1401 digitizing converter (CED) and bandpass filtered at 0.1–1 kHz using a 1902 differential AC amplifier (CED). Evaluated parameter of electromyography (EMG) response is the area under the curve (AUC, represented by mV*ms) of integrated (Su et al., 2019). EMG analysis showed that the AUC was calculated within 10 s from the beginning of water delivery.

### Statistical analyses

All data analyses were analyzed by using Prism 7.0 (GraphPad Software). Sigmaplot (version14.0) and BioRender were used for graphing. Differences among multiple groups were analyzed by one-way ANOVA with Student–Newman–Keuls and two-way ANOVA with Tukey’s multiple comparisons test or Bonferroni’s *post hoc* test. *p* <0.05 was set to indicate statistical significance. Data are represented as mean ± SEM.

### Data availability

The data obtained in this research are available from the corresponding author on reasonable request.

## Results

### EA stimulation regulated the synaptic transmission in the GABAergic neurons, but no effect on tonic inhibition in the excitatory neurons in the contralateral M1

To first assess the effect of stroke and EA treatment on the synaptic activity, considering that the synaptic transmission in the excitatory neurons in the contralateral M1 were demonstrated to be not affected by the stroke in the previous study (L.L. Yao et al., 2022), thus we performed whole-cell patch-clamp recordings of inhibitory neurons in the contralateral M1 from Sham, Stroke, and Stroke + EA groups. We recorded the sEPSCs/sIPSCs in the inhibitory neurons using GAD67-GFP transgenic mice, in which the GABAergic neurons are labeled with GFP (Ono et al., 2005). The results showed that the amplitude of sEPSCs showed no difference among the Sham, Stroke, and Stroke + EA groups (Fig. 1*A*,*B*), while the frequency of sEPSCs in the Stroke group was smaller than that in the Sham group, and EA treatment can rescue this impairment (*p *<* *0.05, Sham: 3.037 ± 0.7459, Stroke: 0.6376 ± 0.1386, Stroke + EA: 2.861 ± 0.7818, Hz; Fig. 1*C*). While the sIPSCs was no altered in the Stroke or Stroke + EA group, comparing to the Sham group (Fig. 1*D–F*). Considering that the extrasynaptic GABA_A_ receptor-mediated tonic inhibition in the excitatory neurons participated in the pathogenesis of stroke (Clarkson et al., 2010), we next validated whether the tonic inhibition was changed in the M1 contralateral to the ischemic brain regions. We found that the holding current induced by PTX (100 μm) was not affected by stroke or EA treatment, suggesting the tonic inhibition in the excitatory neurons in the contralateral hemisphere might not be involved in the stroke pathogenesis or EA treatment (Fig. 1*G*,*H*). Together, these results suggested that the excitatory synaptic transmission in the inhibitory neurons was impaired in the stroke, and this disturbance could be rescued by EA treatment, while tonic inhibition in the excitatory neurons in the contralateral regions might be not involved in the stroke or EA treatment.

### Removal of PNNs in the contralateral M1 affected the EA-mediated regulation of neuronal activity both *in vitro* and *in vivo* in the stroke

**Figure 2.**
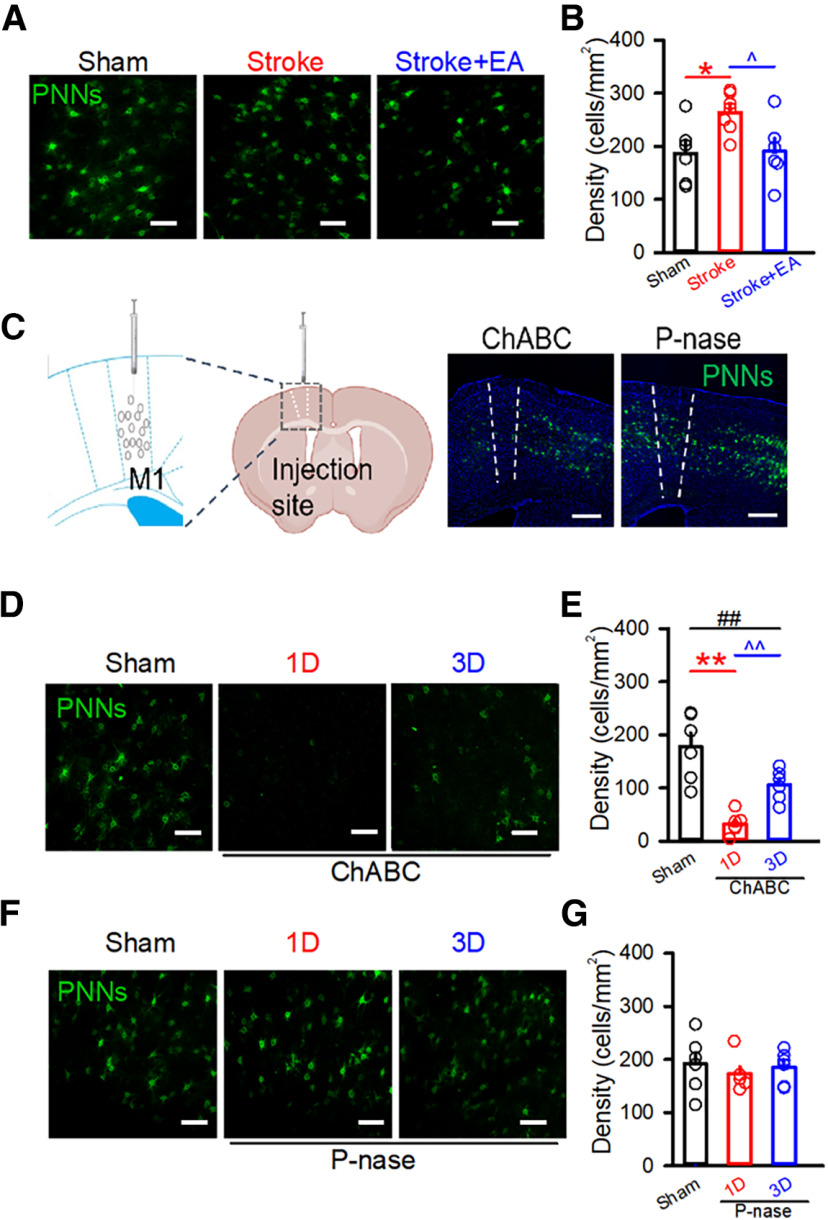
Expression of PNNs was significantly decreased at 1 and 3 d postinjection of ChABC. ***A***, Representative confocal images of PNNs in the Sham, Stroke, and Stroke + EA group. Scale bars: 100 μm. ***B***, Quantification of the density of PNNs in the different groups. The expression of WFA-positive PNNs was significantly increased in the Stroke group, and reduced in the Stroke + EA group. *N* (slices) = 6–7 per group. *N* (mice) = 3 per group. Compared with the Sham group, *p <* *0.05; Compared with the Stroke group, ^*p *<* *0.05. ***C***, Left, The atlas of the mouse brain to show the tip points of injection needles. Right, Representative confocal images showed the injection site of the ChABC or P-nase in the M1. The dashed line was drawn at the site where the drug was injected and the ablation of the PNNs was shown in the ChABC-injected mice. Scale bars: 200 μm. ***D***, Representative confocal images of PNNs in the Sham and ChABC-injected (300 nl, 40 U/ml) group. Scale bars: 100 μm. ***E***, Quantification of the density of PNNs in the Sham and the ChABC-injected group. A significant decrease in the expression of WFA-positive PNNs was significantly decreased in the first day postinjected ChABC group (1 d) compared with the Sham group and the third day postinjected ChABC group (3 d), while the expression of PNNs was still less than that in the Sham group. *N* (slices) = 6 per group. *N* (mice) = 3 per group, respectively. Compared with the Sham group, **p <* *0.01, ##*p *<* *0.01; compared with the 1 d group, ^^*p *<* *0.01. ***F***, Representative confocal images of PNNs in the Sham and P-nase (as a control for ChABC)- injected (300 nl, 40 U/ml) group. Scale bars: 100 μm. ***G***, Quantification of the density of PNNs in the Sham and the P-nase-injected group. The density of PNNs was affected in the P-nase-injected mice at neither first nor third postinjection. *N* (slices) = 6–10 per group. *N* (mice) = 4, 3, and 3 for the Sham, 1 d, and 3 d groups, respectively. Data are shown as mean ± SEM. One-way ANOVA.

Compelling evidence suggested the importance of contralateral brain regions during stroke rehabilitation (Guo et al., 2021; Tang et al., 2022). We first examined the expression of PNNs by immunofluorescence, and the results showed that the expression of PNNs was increased in the stroke condition, while the alteration was reduced after EA treatment (Fig. 2*A*,*B*). The results indicated the therapeutic role of EA might be attributed to the modification of PNNs. To further investigate the role of PNNs in the stroke pathology, the contralateral M1 was pretreated with ChABC, an extensively used enzyme for degrading the PNNs (Fig. 2*C*; Soleman et al., 2012; Banerjee et al., 2017). We first tested the efficacy of injection of ChABC into the M1 to the expression of PNNs. Consistent with the previous studies, the density of PNNs was significantly decreased when the contralateral M1 was injected ChABC 1 d (*p *<* *0.01, Sham: 192.1 ± 11.89, 1 d postinjected ChABC: 32.96 ± 8.237, cells/mm^2^), and this effect was still present but weakened after injecting 3 d (*p *<* *0.01, 1 d postinjected ChABC: 32.96 ± 8.237, 3 d postinjected ChABC: 105.8 ± 11.8, cells/mm^2^; Fig. 2*D*,*E*). However, the density of PNNs was not changed in the P-nase (as a control enzyme for ChABC)-injected group (Fig. 2*F*,*G*). The ChABC eliminated almost all PNNs at 1 d after injection and a recovery of expression was observed after 3 d. In this case, we performed experiment 1 d after ChABC treatment.

**Figure 3.**
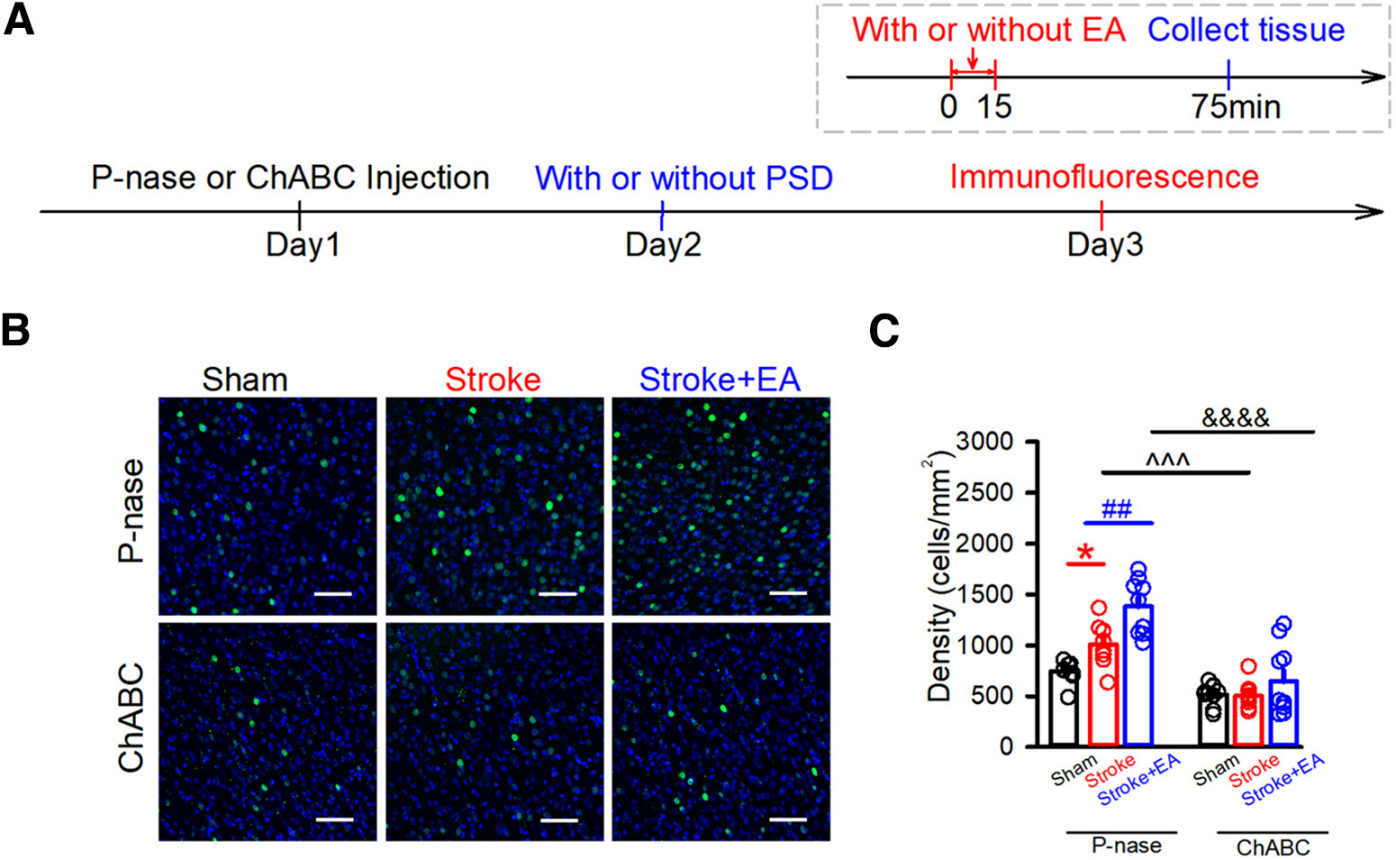
Removal of PNNs in the contralateral M1 affected the expression of c-Fos. ***A***, Schematics of experimental design. ***B***, Representative images showing the expression of c-Fos after P-nase injection or ChABC injection into the contralateral M1 of mice in the Sham, Stroke, and Stroke + EA treatment. Scale bars: 50 μm. ***C***, Quantification of the density of c-Fos expression among three groups after P-nase or ChABC injection into the contralateral M1. The results showed that stroke induction and EA stimulation can induce an enhancement of c-Fos expression in the P-nase-injected group. However, the expression of c-Fos showed no change in the ChABC-injected group. The expression of c-Fos showed a significant decrease in the ChABC-injected mice with stroke induction compared with that in the P-nase-injected mice with stroke induction, and this is the case between the ChABC-injected and P-nase-injected mice with stroke induction and EA treatment. *N* (slices) = 8–10 per group. *N* (mice) = 3 per group. Compared with the Sham group, *p < 0.05; Compared with the Stroke group, ##*p *<* *0.01, ^^^*p *<* *0.001; compared with the Stroke + EA group, &&&&*p *<* *0.0001. Data are shown as mean ± SEM. Two-way ANOVA.

Furthermore, we next determined whether removal of PNNs in the contralateral M1 altered the neuronal activation. We first detected the expression of c-Fos (a marker for activated neurons) in the contralateral M1 by immunofluorescent staining. Interestingly, under the condition of injecting the ChABC, the enhancement of c-Fos expression was absent after stroke or EA treatment (*p *<* *0.01, respectively, Stroke + P-nase: 950.5 ± 91.36, Stroke + ChABC: 502.9 ± 38.36, Stroke + EA + P-nase: 1382 ± 90.87, Stroke + EA + ChABC: 643.6 ± 107.8, cells/mm^2^; Fig. 3*A–C*). However, Stroke induction and EA treatment increased the density of c-Fos compared with the Sham in the P-nase-injected mice (*p *<* *0.05 and *p *<* *0.01, respectively, Sham:749.8 ± 37, Stroke: 950.5 ± 91.36, Stroke + EA: 1382 ± 90.87, cells/mm^2^).

**Figure 4.**
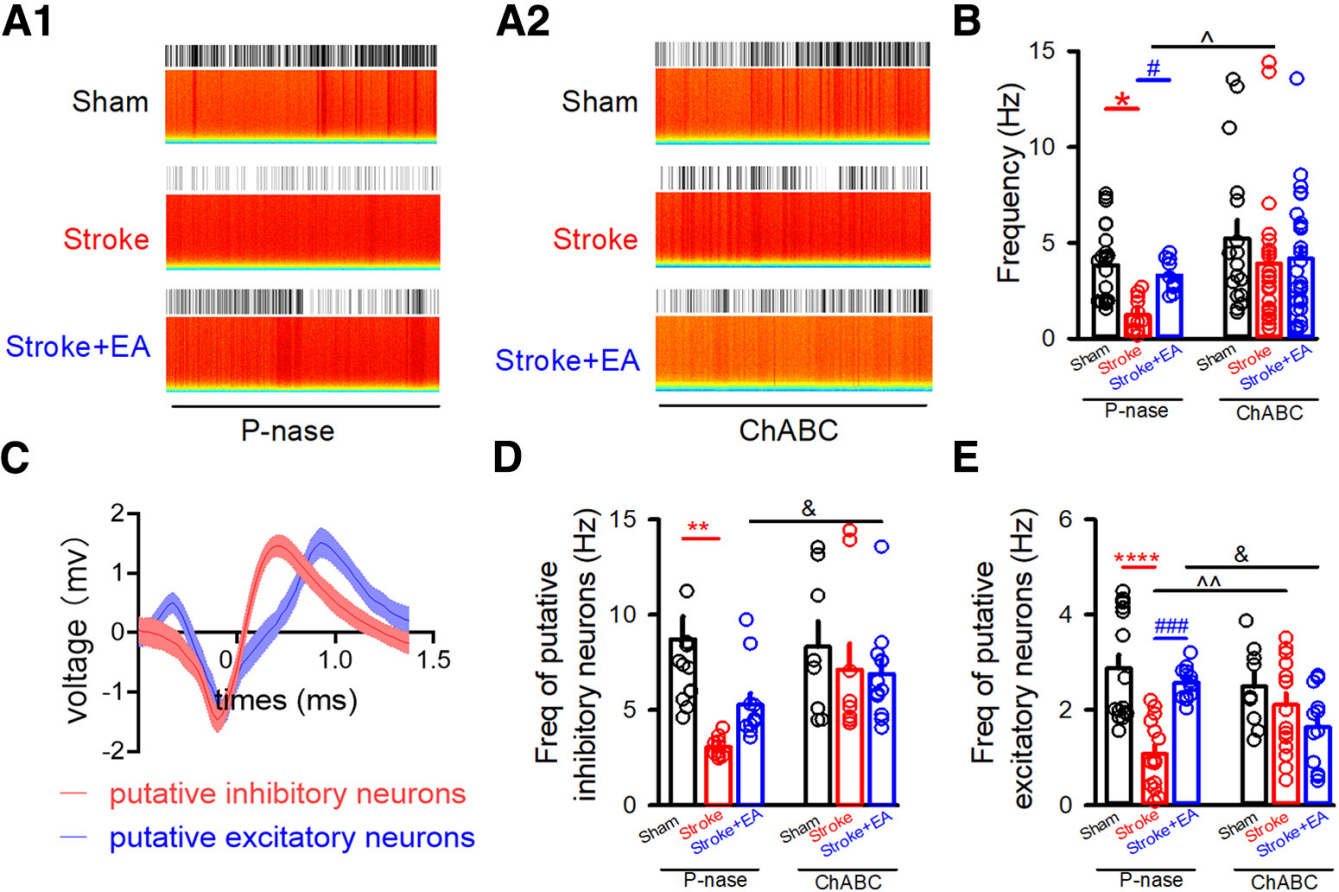
Effects of removal of PNNs to neuronal activity in the contralateral M1 *in vivo*. ***A1***, Example raster and spectrum for spike firing displaying activity at three different conditions in the P-nase-injected mice. The total time of represented raster and spectrum is 300 s. ***A2***, Example raster and spectrum for spike firing displaying activity at three different conditions in the ChABC-injected mice. The total time of represented raster and spectrum is 300 s. ***B***, The spike frequency was impaired in the stroke condition but was rescued by EA treatment in the P-nase-injected mice. There was no significant decrease in spike frequency among the groups after PNNs ablation by ChABC. The spike frequency showed a significant increase in the ChABC-injected mice with stroke induction compared with that in the P-nase-injected mice with stroke induction, but there is no effect between the ChABC-injected and P-nase-injected mice with stroke induction and EA treatment. P-nase-injected group: *N* (units) = 23, 12, and 10 for the Sham, Stroke, and Stroke + EA groups, respectively. ChABC-injected group: *N* (units) = 17, 26, and 26 for the Sham, Stroke, and Stroke + EA groups, respectively. *N* (mice) = 4, 3, and 3 for the Sham, Stroke, and Stroke + EA groups, respectively. Compared with the Sham group, *p < 0.05; compared with the Stroke group, #*p *<* *0.05, ^*p *<* *0.05. ***C***, The representative action potential waveforms of the putative excitatory and inhibitory neurons. ***D***, The frequency of putative inhibitory neurons was impaired in the stroke condition in the P-nase-injected mice, and there was no significant decrease in the frequency of putative inhibitory neurons among the groups after PNNs ablation by ChABC. The frequency of putative inhibitory neurons showed a significant increase in the ChABC-injected mice with stroke induction and EA treatment compared with that in the P-nase-injected mice with stroke induction and EA treatment. P-nase-injected group: *N* (units) = 14, 10, and 11 for the Sham, Stroke, and Stroke + EA groups, respectively. ChABC-injected group: *N* (units) = 8, 9, and 12 for the Sham, Stroke, and Stroke + EA groups, respectively. *N* (mice) = 4, 3, and 3 for the Sham, Stroke, and Stroke + EA groups, respectively. Compared with the Sham group, **p < 0.01; compared with the Stroke + EA group, &*p *<* *0.05. ***E***, The frequency of putative excitatory neurons was impaired in the stroke condition but was rescued by EA treatment in the contralateral M1 in the P-nase-injected mice. However, there was no significant decrease of frequency in putative excitatory neurons among the groups after PNNs ablation by ChABC. The frequency showed a significant increase in the ChABC-injected mice with stroke induction compared with that in the P-nase-injected mice with stroke induction, but there was a similar effect between the ChABC-injected and P-nase-injected mice with stroke induction and EA treatment. P-nase-injected group: *N* (units) = 17, 15, and 13 for the Sham, Stroke, and Stroke + EA groups, respectively. ChABC-injected group: *N* (units) = 9, 16, and 12 for the Sham, Stroke, and Stroke + EA groups, respectively. *N* (mice) = 4, 3, and 3 for the Sham, Stroke, and Stroke + EA groups, respectively. Compared with the Sham group, ****p < 0.0001; Compared with the Stroke group, #*p *<* *0.05, ^^*p *<* *0.01, ###*p *<* *0.001; compared with the Stroke + EA group, &*p *<* *0.05. Data are shown as mean ± SEM. Two-way ANOVA.

Then, to directly explore the role of PNNs in the regulation of neuronal activity in the stroke or EA treatment, *in vivo* electrophysiological recording was used to examine whether the firing rate of neurons in layers 5/6 was affected by the removal of PNNs. Previous studies had shown that impairment of spike frequency in layers 5/6 of contralateral M1 induced by forelimb region of M1, and ischemia stroke was rescued by EA stimulation at Baihui and Dazhui acupoint (L.L. Yao et al., 2022). In the present study, we recorded neurons in layers 5/6 of M1, spike firing in the M1 was decreased by stroke and alleviated by EA treatment in the P-nase-injected mice (Fig. 4*A1*,*B*; *p *< 0.05, respectively, Sham: 4.266 ± 0.4237, Stroke: 1.688 ± 0.2554, Stroke + EA group: 2.955 ± 0.189, Hz). However, the neuronal activity in M1 was not altered by stroke induction or EA treatment in the ChABC-injected mice (Fig. 4*A2*,*B*). While the neuronal spike firing in the M1 was increased by stroke in the ChABC-injected mice compared with that in the P-nase-injected mice (*p *<* *0.05, Stroke+ P-nase: 1.688 ± 0.2554, Stroke + ChABC: 3.927 ± 0.6704, Hz). We further analyzed the neuronal type obtained from *in vivo* electrophysiological recording. The results showed that the frequency of putative excitatory and inhibitory neurons was reduced in the Stroke group, while the frequency of putative excitatory neurons in the P-nase-injected mice was rescued by EA treatment, and these alterations were disappeared in the ChABC-injected-mice (Fig. 4*C–E*).

**Figure 5.**
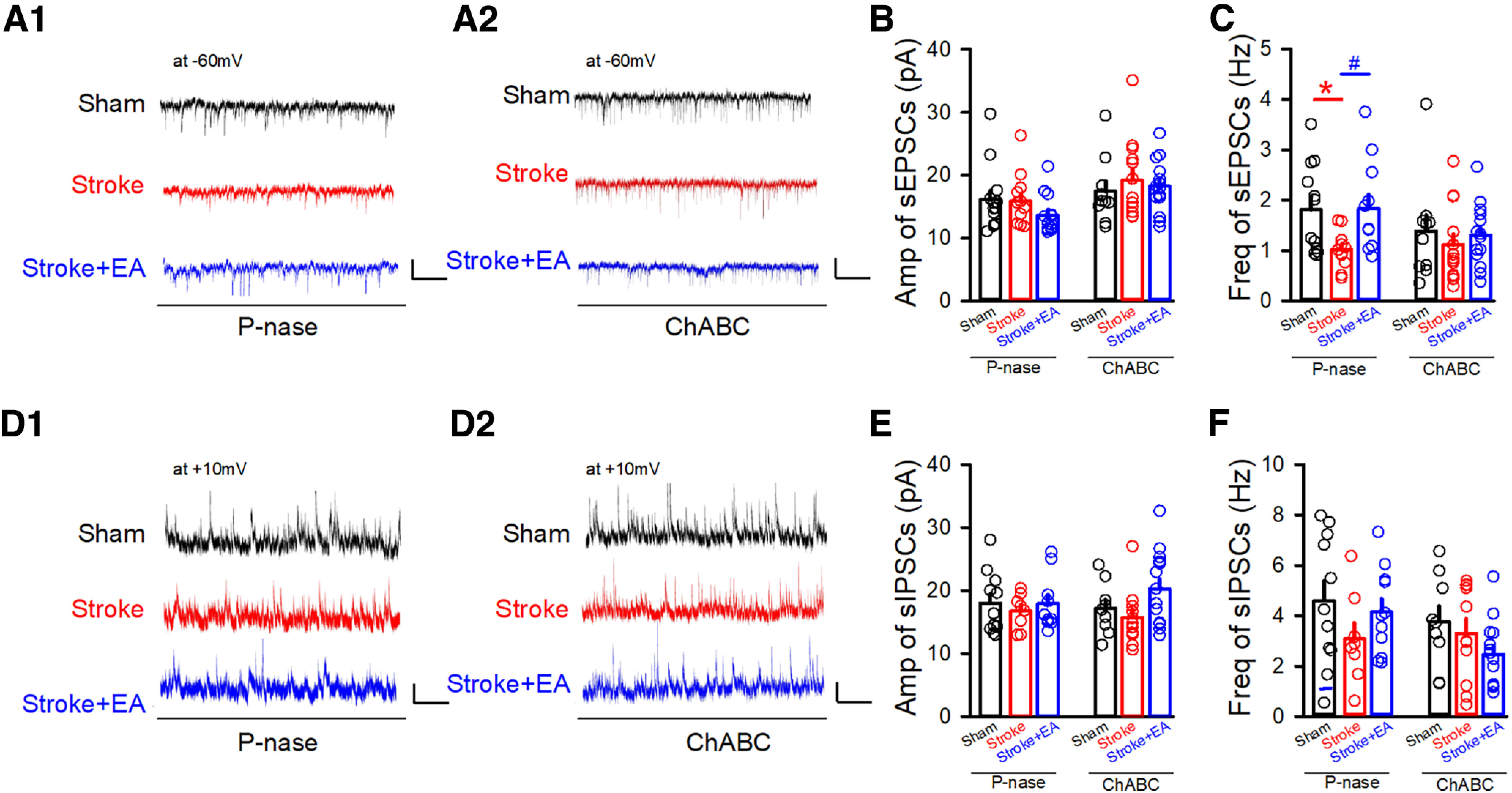
Effects of removal of PNNs to sEPSCs in the excitatory neurons *in vitro*. ***A1***, Sample traces showing sEPSCs recorded in the excitatory neurons clamped at −60 mV in the P-nase-injected mice. Scale bars: 1 s, 20 pA. ***A2***, Sample traces showing sEPSCs recorded in the excitatory neurons clamped at −60 mV in the ChABC-injected mice. Scale bars: 1 s, 20 pA. ***B***, Quantifications of amplitude (Amp) in the contralateral M1 from Sham, Stroke, and Stroke + EA groups in the P-nase or ChABC-injected mice. The results showed that the amplitude of sEPSCs were no altered in the P-nase-treated or ChABC-treated mice. *N* (cells) = 10–14 per group. P-nase-injected group: *N* (mice) = 6, 4, and 3 for the Sham, Stroke, and Stroke + EA groups, respectively. ChABC-injected group: *N* (mice) = 6, 3, and 3 for the Sham, Stroke, and Stroke + EA groups, respectively. ***C***, Quantifications of frequency (Freq) in the contralateral M1 from Sham, Stroke, and Stroke + EA groups in the P-nase-injected or ChABC-injected mice. The results showed that stroke affected the frequency of sEPSCs, and this impairment could be improved by EA treatment in the P-nase-injected mice, while the frequency of sEPSCs in the excitatory neurons showed no difference in the ChABC-injected mice. *N* (cells) = 10–14 per group. P-nase-injected group: *N* (mice) = 6, 4, and 3 for the Sham, Stroke, and Stroke + EA groups, respectively. ChABC-injected group: *N* (mice) = 6, 3, and 3 for the Sham, Stroke, and Stroke + EA groups, respectively. Compared with the Sham group, *p < 0.05; compared with the Stroke group, #*p *<* *0.05. ***D1***, Sample traces showing sIPSCs recorded in the excitatory neurons clamped at +10 mV in the P-nase-injected mice. Scale bars: 1 s, 20 pA. ***D2***, Sample traces showing sIPSCs recorded in the excitatory neurons clamped at +10 mV in the ChABC-injected mice. Scale bars: 1 s, 20 pA. ***E***, Quantifications of amplitude (Amp) in the contralateral M1 from Sham, and Stroke, and Stroke + EA groups in the P-nase or ChABC-injected mice. The results showed that the amplitude of sIPSCs were no longer influenced in the P-nase-treated or ChABC-treated mice. *N* (cells) = 8–15 per group. P-nase-injected group: *N* (mice) = 6, 4, and 3 for the Sham, Stroke, and Stroke + EA groups, respectively. ChABC-injected group: *N* (mice) = 6, 3, and 3 for the Sham, Stroke, and Stroke + EA groups, respectively. ***F***, Quantifications of frequency (Freq) in the contralateral M1 from Sham, Stroke, and Stroke + EA groups in the P-nase or ChABC-injected mice. The results showed that the frequency of sIPSCs were no longer influenced in the P-nase-treated or ChABC-treated mice. *N* (cells) = 8–15 per group. P-nase-injected group: *N* (mice) = 6, 4, and 3 for the Sham, Stroke, and Stroke + EA groups, respectively. ChABC-injected group: *N* (mice) = 6, 3, and 3 for the Sham, Stroke, and Stroke + EA groups, respectively. Data are shown as mean ± SEM. Two-way ANOVA.

For the excitatory neurons constitutes ∼80% of the total neurons in the cerebral cortex (Markram et al., 2004), we mainly tested the effect of removal of PNNs to excitatory neurons *in vitro*. The slice electrophysiological recording was adopted to record the synaptic transmission in the excitatory neurons. In the P-nase-treated mice, the amplitude of sEPSCs in the excitatory neurons showed no difference among the three groups, including Sham, Stroke, and Stroke + EA groups, while there was a significant decrease of frequency of sEPSCs in the Stroke group compared with that in the Sham group, and this impairment could be improved by EA treatment (Fig. 5*A–C*; *p *<* *0.05, respectively, Sham group: 1.741 ± 0.248, Stroke group: 1.07 ± 0.1146, Stroke + EA group: 1.679 ± 0.2561, Hz). However, the frequency of sEPSCs in the ChABC-treated mice was no longer altered in the Stroke or Stroke + EA group. Moreover, the amplitude and frequency of sIPSCs did not differ among the Sham, Stroke, and Stroke + EA groups (Fig. 5*D–F*). These results suggested that the lower synaptic density in the excitatory neurons and/or a lower probability of glutamate was released from the presynaptic terminals connecting to postsynaptic excitatory neurons in the Stroke, and this could be recovered by EA treatment. In summary, all these results indicated that the PNNs in the contralateral M1 might be crucial for modulating neuronal activity during stroke pathogenesis or EA treatment.

### The EA-mediated improvement of swallowing function was prevented by removing the PNNs in the contralateral M1

**Figure 6.**
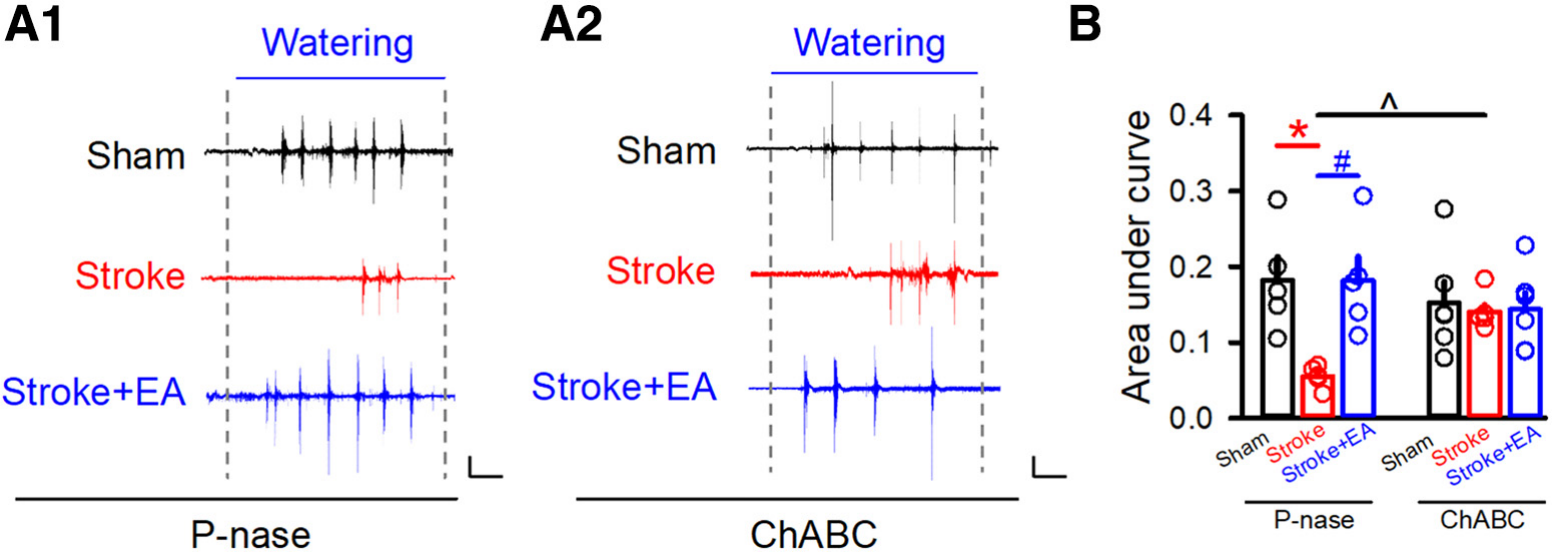
The dysfunction of swallowing function in stroke or the effect of EA disappeared after the removal of PNNs. ***A1***, The representative traces of EMG at the mylohyoid in the P-nase-injected mice in the Sham, Stroke, and Stroke + EA groups, respectively. Scale bars: 2 s, 0.1 mV. ***A2***, The representative traces of EMG at the mylohyoid in the ChABC-injected mice in the Sham, Stroke, and Stroke + EA groups, respectively. Scale bars: 2 s, 0.1 mV. ***B***, Quantification of the area under the curve of EMG from every group. *N* (mice) = 5–6 per group. The results showed that EA could rescue the impaired swallowing function induced by stroke, and the phenomenon disappeared when PNNs were removed by ChABC, while a significant increase in the ChABC-injected mice with stroke induction compared with that in the P-nase-injected mice with stroke induction. Compared with the Sham group, *p <* *0.05; compared with the Stroke group, #*p *<* *0.05, ^*p *<* *0.05. Data are shown as mean ± SEM. Two-way ANOVA.

We next aimed to validate the function of PNNs in the EA-mediated improvement of swallowing function. EMG recording in the mylohyoid was detected after P-nase or ChABC injection. We found that EA could enhance the impaired swallowing function induced by Stroke in the P-nase-injected mice (*p *<* *0.05, respectively, Sham: 0.1683 ± 0.02,857, Stroke: 0.07398 ± 0.008731, Stroke + EA: 0.1649 ± 0.03,064, mV*ms; Fig. 6*A1*,*B*). However, the Stroke-induced impairment and EA-induced enhancement was absent in the groups with the removal of PNNs by ChABC (*p *<* *0.05, Stroke + ChABC group: 0.1197 ± 0.006308, Stroke + P-nase group: 0.07398 ± 0.008731, mV*ms; Fig. 6*A2*,*B*). The swallowing function was recovered during Stroke condition in the ChABC-injected mice compared with the P-nase-injected mice, but there was no more improvement of EA treatment. Overall, these results indicated that PNNs might be involved in the process of Stroke pathogenesis and EA-mediated improvement of swallowing function.

## Discussion

In the present study, we found that the spontaneous excitatory synaptic transmission in the inhibitory neurons in the contralateral M1 was impaired in the stroke and potentiated by EA treatment. While disturbing the PNNs in the contralateral hemisphere with pretreatment of ChABC could prevent the M1 ischemia-induced abnormal c-Fos expression, impaired neuronal activity *in vivo* and *in vitro*, and dysfunction of swallowing function, and the EA efficacy was also disappeared. Our study provides insight that PNNs in the contralateral M1 might involve in stroke pathology and EA treatment for stroke rehabilitation.

In the brain, ischemic stroke results in local cell death and disruption of both local and remote functional neuronal networks (Quattromani et al., 2018a). Neuroplasticity and reorganization of networks are thought to contribute to stroke recovery (Katarzyna Greda and Nowicka, 2021). Previous studies have demonstrated that the PNNs removal by ChABC reactivated neuroplasticity and promoted sensorimotor recovery after stroke (Soleman et al., 2012; Madinier et al., 2014). Additionally, removal of PNNs in the spinal cord after injury enhances motor recovery (Irvine and Kwok, 2018). Modulating the PNNs after ischemic damage may provide new therapies enhancing tactile proprioceptive function after stroke (Madinier et al., 2014). In the present study, ChABC was administered with pretreatment before stroke induction and injected locally into the contralateral M1 to degrade the PNNs. The ChABC treatment eliminated almost all *Wisteria floribunda* agglutinin (WFA) signals at 1 d after injection and a gradual recovery in WFA intensity was observed after 3 d. The results demonstrated that removal of PNNs 1 D after ChABC treatment attenuated the dysfunction of the swallowing process, c-Fos expression, or neuronal activity, and the EA efficacy was disappeared as well. These data should be interpreted with caution because that PNNs degradation by ChABC itself might promote stroke recovery, thus the effect of EA might be masked, which is a limitation of the study. To this end, the modulators exogenously upregulating the PNNs expression should be applied; however, these modulators upregulating the PNNs are not available currently. Although, the hypothesis of PNNs involving the EA-mediated treatment for stroke improvement was suitable for accounting the observation, for the recovery showed no better after EA treatment with ChABC. Therefore, our study suggested that the PNNs in the contralateral M1 was associated with the rehabilitation of PSD mice and suggested to participate in the pathogenesis. However, whether PNNs in the contralateral M1 participated in the regulation of EA-mediated swallowing function in PSD mice still needs to be investigated.

Previous studies showed that the density of PNNs was decreased in the degenerating lesion core, followed by the reduction in perilesional and remote areas as well as in homotopic contralateral regions (Karetko-Sysa et al., 2011; Alonge et al., 2021). In the ischemic core, and to some extent in the perilesional area, excitotoxic events take place, including the rapid increase in intracellular calcium and activation of many calcium-dependent proteases resulting in cell death (Karetko-Sysa et al., 2011). The proteases, such as tPA, ADAMTS-4, MMP-2, and MMP-9, released from heavily damaged cells contribute to PNN disintegration. (Karetko-Sysa et al., 2011). The evidence from the stroke patients also demonstrated that the expression of PNNs and the related proteases or inhibitor was altered in the ipsilateral hemisphere. (Quattromani et al., 2018b). In the study, we only detected the role of PNNs in the contralateral M1, the role of PNNs in the ipsilateral M1 needs to be detected in the future. Meanwhile, it is noteworthy that some evidence obtained from the quadruple knockout mouse lacking the ECM components worsen the behavioral outcome in the early stage of stroke, while subsequently facilities the functional recovery (Quattromani et al., 2018a). Thus, the role in the different time point after a stroke needs to be dug out in additional experiments.

PNNs play an important role in regulating synaptic functions and plasticity, and genetic or enzymatic reduction of PNNs can increase synaptic transmission (Reichelt et al., 2019). Previous studies showed that 7 d after ChABC treatment reduced both intrinsic excitability of parvalbumin neurons (PV^+^) and synaptic transmission to both PV^+^ neurons and excitatory neurons in the primary visual cortex, but 1 d after ChABC treatment digested PNNs effectively but had no effects on intrinsic excitability or synaptic transmission (Liu et al., 2022). Other similar studies showed that acute ChABC treatment showed no effect, while long-term treatment impacted the synaptic transmission and intrinsic excitability of PV^+^ neurons (Shi et al., 2019), and no changes in intrinsic properties or sEPSCs/sIPSCs in the principal neurons after ChABC (2∼3 d) treatment in the visual cortex (Faini et al., 2018). Therefore, removing PNNs *in vivo* using ChABC led to distinct changes in neuronal excitability and synaptic transmission, depending on different time courses of ChABC digestion (Liu et al., 2022). In the present study, we found no significant changes of synaptic transmission in the excitatory neurons between the P-nase and ChABC treated groups, which might be attributed with the interval of time after removal the PNNs not enough long to take the effect (Senkov et al., 2014). Thus, the effect of different interval of time after removal of the PNNs to the synaptic transmission needs to be further explored in the future study. Meanwhile, a common finding in many parts of the brains is that PNNs depletion decreases GABAergic inhibition, possibly indicating that PNNs act to limit the numbers of inhibitory synapses that form on PV^+^ interneuron (Fawcett et al., 2019). The study demonstrated that the frequency of sEPSCs or sIPSCs decreased with no change of the amplitude, suggesting a decrease in the number of excitatory synaptic inputs apposing PV interneurons (Favuzzi et al., 2017). The relationship between the PNNs and PV interneurons or other inhibitory neuronal types is also worthy to be studied.

Although we have unraveled that the PNNs are associated with the stroke pathology and might be involved the EA-mediated function recovery, the mechanism underlying this process is still not be elucidated. PNNs may convey molecular signals by binding and storing proteins with important roles in cellular communication (Boggio et al., 2019). Semaphorin3A (Sema3A), as an essential member of the semaphore family, is mainly synthesized by neurons (Williams et al., 2007). Sema3A may be an important functional component of PNNs in the adult brain and accumulate in PNNs in an experience-dependent manner, and its presence in the cortex inhibits plasticity (Boggio et al., 2019). It has been reported that PNNs have been linked to the transcription factor orthodenticle homeobox protein 2 (OTX2; Harkness et al., 2021). Specific transfer of OTX2 homeoprotein into interneurons is a critical period of plasticity in the cortex (Beurdeley et al., 2012). EA may affect the plasticity of PNNs by regulating the Sema3A signal and promoting the recovery of the motor function after spinal cord injury (Hu et al., 2020), but whether EA affect the PNNs by regulating the OTX2 is unknown. It is worthwhile that other critical period signaling molecules, including neuronal activity-regulated pentraxin (Narp) and Nogo receptor (NgR), are required for PNNs to function as a brake on plasticity (Takesian and Hensch, 2013). There are few kinds of literature on exploring the role of PNNs in the EA-mediated treatment for stroke rehabilitation. As limited studies explore the mechanism underlying the EA treatment, more studies on how the current topic of EA treatment regulating the PNNs to promote stroke recovery is encouraged to uncover this puzzle.

In the study, we examined whether EA at the CV23 acupoints activated M1 neurons by assessing the expression of c-Fos and electrophysiological recording. In the mice with injecting the P-nase, the expression of c-Fos was increased (Fig. 3*B1*,*C*), while the neuronal activity represented by spike spiking was decreased by stroke induction (Fig. 4*A1*,*B*). The similar result has been observed in the previous published study that changes in c-Fos were observed but no changes in neuronal activity were observed by in *vivo* electrophysiological recording (S. Yao et al., 2020). Although c-Fos, an intermediate early gene that serves as a molecular marker of neuronal activation (Ivashkina et al., 2021), it is expressed only in some kinds of neurons following peripheral stimulation, which might be inducible only in neurons that contain a particular neurotransmitter, that produce a particular kind of action potential, or that possess particular kinds of receptors. Therefore, it may be an incomplete marker for responsive activity (Bullitt, 1990). The presence of neuronal labeling may be interpreted as indicative of neuronal activation, while the absence of such labeling cannot be used to exclude the possibility of neuronal excitation (Bullitt, 1990). For the neuronal activity represented by spike firing for the *in vivo* electrophysiological recording, this could be influenced by the general condition of the mice, such as anesthetic level or exogeneous spontaneous stimulus (Haumesser et al., 2017; Duchamp-Viret and Chaput, 2018; Fitzgerald and Watson, 2019). Additionally, we used brain slices recordings to detect neuronal activity, but the amplitude and frequency of sIPSCs showed no alteration in the stroke. That is because that the neuronal activity was determined by two factors, synaptic input and intrinsic excitability (van Vreeswijk and Sompolinsky, 1996). The data from *in vivo* electrophysiological recording (Fig. 4) demonstrated a decreased neuronal activity, which may be mostly attributable to impaired synaptic input. However, most of these inputs have been disconnected *in vitro*, and the intrinsic excitability might be mainly contributed to the results of sIPSCs (Figs. 1 and 5). Thus, it should be paid more attention when made a conclusion on the neuronal activity induced by stroke or EA treatment. By the way, in these electrophysiological recording and immunofluorescence experiments, lack of the behavioral tests evaluating the PSD model at baseline is also a limitation of the present study.

## Synthesis

Reviewing Editor: Francisca Bronfman, Universidad Andres Bello Facultad de Medicina Decisions are customarily a result of the Reviewing Editor and the peer reviewers coming together and discussing their recommendations until a consensus is reached. When revisions are invited, a fact-based synthesis statement explaining their decision and outlining what is needed to prepare a revision will be listed below. The following reviewer(s) agreed to reveal their identity: NONE.

The manuscript investigating synaptic mechanism of EA-induced effect on post-stroke dysphagia was evaluated by two reviewers who have expertise in the field. Both reviewers found merits of the study including expertise in the application of EA, electrophysiological mechanisms to assess the efficacy, and an involvement of contralateral side for the effects. However, the reviewers also raised several concerns related to the lack of immunohistochemical staining and statistical analyses. Detailed and specific comments from the reviewers are appended.

Reviewer 1

In this paper, the authors investigated the underlying mechanisms of therapeutic effects of EA at Lianquan (CV23) on improved swallowing function in PSD. They found EA recovered the impaired sEPSCs in the GABAergic neurons in the contralateral M1. Further, they found EA induced increase of c-Fos expression, enhancement of spike firing, potentiation of sEPSCs in the excitatory neurons, and the improved swallowing function were all blocked by the removal of PNNs in the contralateral M1. The authors have strong experiences working with EA in stroke and did a thorough and detailed discussion. Clarification of issues below would strengthen the manuscript.

Major points:

1. In Figure 5, the authors showed that the swallowing function was recovered during stroke condition in the ChABC-injected mice compared to the P-nase-injected mice, but there was no more improvement of EA treatment. To clarify the therapeutic role of EA via modifying PNNs, would you please provide the direct evidence by immunostaining of PNNs in the sham, stroke (contralateral side) and EA (contralateral side treatment) group?

2. The authors provided the data presented in Figure 1 and Figure 4, which demonstrated that both excitatory and inhibitory neurons had decreased pre-synaptic excitatory input and rescued by EA treatment measured by in vitro brain slices recording. In Figure 3, would you please clear what kind of neuronal type was represented, excitatory or inhibitory? According to identified well-isolated units in each group (Figure 3B), how many units were putative excitatory or inhibitory neurons? If it is possible, would you please display representative action potential waveforms of the putative excitatory and inhibitory neurons?

3. In Figures 2C, 3B, 4B-C, 4E-F, 5B, please use two-way ANOVA.

Minor point:

1. Typos: Line 319, ChABC-injeted or ChABC-injected?

Reviewer 2

It is a new field and interesting for the acupuncture research,and provides new evidence from the perspective of in vitro slice electrophysiological recording, in vivo electrophysiological recording, and immunofluorescent staining. The manuscript is relatively clear. However, there are some points need to be noted.

1. It’s better to add a reference about the location of acupoint of CV23.

2. For Table 2 Abbreviations, it should be listed in the order of alphabet.

3. It’s better to add “Supplementary figures” into the main text.

4. The figures of immunofluorescence staining are too small to visualize. If is possible, instead of DAPI, it’s better to use NeuroTraceTM Fluorescent Nissl Stain for tissue counter staining, which can reflect the cortical neurons more clearly.

## Author Response

Dear editor and reviewers,

We appreciated the critical and thoughtful comments and suggestions from the reviewer to improve our manuscript. Accordingly, we answered questions from the reviewers.

Please find our detailed point-by-point responses below.

Sincerely,

Lulu Yao

-

Point-by-point responses (MS ID: eN-NWR-0234-23)

Comments:

1. C-fos is usually used as a marker for increased neuronal activity as the authors’ purpose. Would you please explain why stroke can induce increased c-fos expression (Figure 3B-C), but decrease the frequency of both putative excitatory and inhibitory neurons (Figure 4)?

Response: Thanks for your pointing out this issue. The spike firing obtained by in vivo electrophysiological recording was collected in the mice with spontaneous free moving (Figure 4), thus the mice with stroke condition potentially less influenced by the exogeneous stimulus from the environment showed a decrease of neuronal activity. While the increased expression of c-Fos stroke induction within 24 h indicated these neurons in this brain region were activated at this time point, as also demonstrated by other studies (PMID: 24434515, 10375656). Moreover, this inconsistence has been also reported in other studies (PMID: 32519209). These points have been discussed in the manuscript (Pages 19-20, Line 412-427).

2. Would you please explain the differences between brain slices recordings and in vivo recording data regarding inhibitory transmission? In Figures 1 and 5, stroke has no effect on the amplitude and frequency of sIPSC. In Figure 4, stroke can decrease the frequency of putative inhibitory neurons.

Response: The neuronal activity was determined by two factors, synaptic input and intrinsic excitability (PMID: 8939866). The data from in vivo electrophysiological recording (Figure 4) demonstrated a decreased neuronal activity, which may be mostly attributable to impaired synaptic input. As mentioned above, the mice with stroke condition potentially less influenced by the exogeneous stimulus from the environment showed a decrease of neuronal activity. However, most of these inputs have been disconnected in vitro, and the intrinsic excitability might be mainly contributed to the results of sIPSCs (Figure 1 and 5), while the intrinsic excitability of neurons located in the contralateral M1 has been demonstrated to be not affected by stroke by brain slice recording (PMID: 34782576). Therefore, the amplitude and frequency of sIPSCs showed no alteration in the stroke (Pages 20, Line 428-434).
